# Interpersonal wounds and identity among people with anorexia nervosa: a qualitative content analysis of autobiographical memories elicited by disgust-related cues

**DOI:** 10.1186/s40337-026-01676-w

**Published:** 2026-06-08

**Authors:** Sevgi Bektas-Dag, Fidan Turk, Hubertus Himmerich, Janet Treasure, Johanna L. Keeler

**Affiliations:** 1 Department of Psychology, Hacettepe University, Ankara, Türkiye; 2 Department of Psychology, University of Exeter, Exeter, UK; 3 Centre for Research in Eating and Weight Disorders (CREW), Institute of Psychiatry, Psychology and Neuroscience, King’s College London, London, UK; 4 Present Address: Department of Child and Adolescent Psychiatry, Psychosomatics and Psychotherapy, LVR University Hospital Essen, University of Duisburg- Essen, Wickenburgstrasse 21, 45147 Essen, Germany

**Keywords:** Episodic memory, Autobiographical memory, Anorexia nervosa, Disgust, Qualitative content analysis

## Abstract

**Background:**

Research on autobiographical memory (AM) in anorexia nervosa (AN) has largely focused on quantitative aspects such as specificity, while qualitative investigations of thematic patterns remain limited. This study aimed to compare the content of cued AMs between individuals with AN and healthy controls (HCs) using a qualitative approach.

**Methods:**

Sixty-three adults (AN = 43; HC = 20) completed a computerised written version of the Autobiographical Memory Test, generating memories in response to six-self- or moral-disgust-related cue-words. A total of 189 AMs (AN = 129; HC = 60) were coded using a conventional qualitative content analysis.

**Results:**

Fourteen codes were constructed from the data, two of which were excluded from theme development. Three overarching themes were identified: (1) relational wounds – the role of others, (2) relational vigilance, and (3) identity disturbance. AMs reflecting *iatrogenic harm*, *body mistrust*, *betrayal*, *impact of interpersonal experiences*, *moral self-evaluation*, *shame*, and *feelings of wrongness or being different* were unique to, or more frequent in, the AN group than HCs. Across themes, individuals with AN exhibited more intense, enduring, and unresolved relational and self-related difficulties, whereas HCs described similar experiences in a more situational, flexible, and adaptive manner.

**Conclusions:**

A qualitative exploration of the content of cued AMs from people with AN is a novel approach to understanding its phenomenology. In this study, interpersonal and self-related mistrust were prominent features of AMs in people with AN. Understanding these qualitative differences in AM content may inform interventions aimed at restoring relational trust and self-acceptance.

**Supplementary Information:**

The online version contains supplementary material available at 10.1186/s40337-026-01676-w.

## Abstract (highlights)

This study utilised a qualitative approach to compare the content of autobiographical memories elicited by self- and moral-disgust-related cues between individuals with anorexia nervosa and healthy controls. Three overarching themes emerged: relational wounds – the role of others, relational vigilance, and identity disturbance. Compared to healthy controls, individuals with anorexia nervosa described more intense, enduring, and unresolved relational and self-related difficulties, whereas healthy controls recalled similar experiences in a more flexible and adaptive manner. Interpersonal and self-related mistrust were prominent features of autobiographical memory content in anorexia nervosa. Autobiographical memory enquiry may constitute a valuable clinical tool, offering insights into the origins of an individual’s difficulties and their effects on current presenting problems, as well as the individual's views of themselves, others, and the world, thereby supporting psychological formulation. These findings also have implications for intervention, pointing to the value of trauma-informed approaches that directly target relational trust and self-acceptance in the context of anorexia nervosa recovery.

**Supplementary Information:**

The online version contains supplementary material available at 10.1186/s40337-026-01676-w.

## Introduction

Anorexia nervosa (AN) is an eating disorder (ED) characterised by persistent restriction of energy intake, significant weight loss, an intense fear of weight gain, and disturbances in the perception of body shape and size [1]. Within the spectrum of EDs, AN is associated with elevated mortality rates, significant medical and psychosocial risks, and marked reductions in quality of life [2]. Although the aetiology of AN remains incompletely understood and is heterogeneous in nature, current evidence suggest that its development likely reflects an interaction of biological, interpersonal, and psychosocial influences [3–5].

Within this broader framework, specific memory deficiencies have been proposed as one factor in the maintenance of and relapse risk for AN [6, 7]. A specific memory system that has received particular attention in this context is autobiographical memory (AM), defined as the recall of personally meaningful past experiences [8], which has been found to be affected in individuals with AN. More specifically, research has suggested that deficiencies in retrieving specific details of AMs (i.e., overgeneral autobiographical memory; OGM) [9] is a state-related aspect of AN psychopathology, observed in individuals with acute AN [10–12]. However, AM abilities may also be influenced by mood-related factors or other symptoms of psychopathology in individuals with AN, as the aforementioned OGM effect has been shown to become non-significant once comorbid depressive symptoms are controlled for [12]. In one of our previous studies [13] that did not find differences in memory specificity between the AN (including atypical AN) and HC groups, higher levels of childhood teasing and betrayal sensitivity predicted greater vividness of memories generated in response to negative cue-words, an effect that did not emerge in the HC group. This finding raised the likelihood that there may be differences between groups in the *content* of memories, not only the ability to recall specific details.

Standard statistical analyses of AM tasks, however, risk overlooking these patterns by focusing on quantitative indices and disregarding the qualitative aspects of memory content. We have in our previous work [12, 14] proposed that autobiographical memory problems or biases may disrupt the understanding of personal narratives and, in turn, adaptive identity formation and integration, which may also be related to prolonged entrenchment in AN. The use of self- or moral-disgust–relevant cue-words (mistrust, disloyalty, exclusion, betrayal, let down, shame) in our prior study allows for a qualitative examination of AMs, shedding light on the salience of these experiences and the meanings individuals ascribe to them, thereby capturing what is recalled rather than merely how specifically the memory is recalled. This is particularly relevant for individuals with AN, who frequently report heightened levels of self-disgust [15], which is likely to persist even after physical recovery [16]. Furthermore, as outlined in the theoretical framework proposed by Glashouwer and de Jong [17], moral disgust, elicited by actual or perceived transgressions of socio-moral norms, has been suggested as another disgust domain implicated in AN psychopathology. Individuals with AN are often highly sensitive to punishment [18] or social rejection [19], and the internalization of rigid socio-moral rules (e.g., notions of “good” or “bad”) may function to reduce the risk of interpersonal rejection within social contexts. Although moral disgust has been primarily discussed in relation to disorder-specific concerns (e.g., body), early adverse life events may render individuals vulnerable to experiencing moral disgust in response to broader situations that resemble past negative experiences. Such processes may contribute to interpersonal difficulties [20, 21], which is also likely to have relevance for the therapeutic alliance.

To the best of our knowledge, no research has examined whether, and in what ways, the qualitative content of AMs reported by individuals with AN differs from that of healthy controls (HC), despite the value of such work for understanding the meaning attributed to and nature of the recall of difficult personal experiences in people with AN. The present study aimed to address this gap in the literature by exploring the content of AMs elicited by self- or moral-disgust-related cue-words to identify both shared and group-specific patterns, which may have implications for the understanding of the phenomenology of AN, and in particular, elicited narratives of specific past events. Given the nature of the data acquired (i.e., short written descriptions of memories elicited during the Autobiographical Memory Test (AMT; [22]), we employed an inductive qualitative content analysis to systematically identify and categorize meaning from the data. A coding framework was first developed from the AN dataset and then applied to the HC dataset to compare emerging patterns across groups.

## Methods

### Participants

Individuals with AN (*n* = 43) were recruited from the South London and Maudsley NHS Trust (SLaM) and recruitment websites (e.g., BEAT). The HC (*n* = 36) group was recruited via email research circulars at King’s College London (KCL) and social media platforms (e.g., LinkedIn, Twitter). To be eligible for the study, participants had to meet the following criteria: a current diagnosis of AN (for the AN group) or no current ED (for HCs), fluency in English and access to a computer with a stable internet connection. Diagnoses of AN were verbally confirmed during a pre-study phone call conducted by SBD, a psychologist with three years of experience working with patients at Department of Psychiatry, Ankara University Faculty of Medicine. During this call, participants confirmed their diagnosis, the professional context in which it was received, and their current or recent treatment status. For HCs, the pre-study call was used to confirm the absence of any current or past eating disorder diagnosis. The Eating Disorder Diagnostic Scale (EDDS; [23, 24]) was administered to all participants to screen for diagnoses of AN and to confirm that HCs did not meet diagnostic criteria for any ED. All participants were also required to have no history of, or current post-traumatic stress disorder (PTSD), substance abuse, psychotic disorders, or neurological disorders, as these psychiatric and neurological conditions are associated with difficulties with AM retrieval [25]. The present qualitative study represents a secondary analysis of a subset of data previously acquired and published by Bektas et al. [13]. This study received ethical approval from the London Bridge NHS Research Ethics Committee (Reference: 18/LO/0121). Informed consent was obtained from all participants using an approved participant information sheet and consent form. Participants were given £10 for their time and consented to their data being used for further analyses. In the initial sample (*n* = 79), many HCs were residing in the UK for postgraduate studies, resulting in a significant difference in ethnic distribution, with non-Caucasian participants being more prevalent in the HC group. Previous evidence suggests that racial/ethnic minority, immigrant, and refugee youth are more likely to have experienced victimisation within school and community contexts [26], suggesting that this ethnic imbalance could potentially influence the content of AMs and represent a confounding factor. To address this, we applied frequency matching: sixteen HC participants were randomly excluded to balance the distribution of ethnic categories across groups.

### Data collection

In the original study [13], participants completed computerised written versions of the AMT [22] and the Episodic Future Thinking Task (EFT-T; [27]) where they were given two minutes to generate a specific event that personally experienced in the past or might experience in the future. This study used the online platforms Qualtrics (www.qualtrics.com) and Gorilla [28] to create and host tasks.

Participants provided written informed consent and received an email where they were instructed on how to create an optimal testing environment, including muting background music, silencing their mobile phones, ensuring they were in a quiet and undisturbed space with a stable internet connection, informing others that they would be unavailable for approximately one hour if necessary, and setting their browser to full-screen mode. They were also informed to give themselves enough time to read instructions carefully during the tasks. The researcher (SBD) was able to monitor participants’ progress through the study platform in real time and remained available via email and phone throughout the session to address any questions or clarify task instructions as needed. At the end of the AMT and EFT-T, a short positive mood induction (3 min) of relaxing music was administered. Participants were also asked to share their experiences of the computer-based tasks to ensure they had not experienced any significant distress and to determine whether any further support was needed, as outlined in the Participant Information Sheet. In addition, participants were reminded that they could pause or discontinue the study at any time if they felt uncomfortable or distressed and were signposted to appropriate mental health support resources if needed.

Instructions at the beginning of the task informed participants that the memory/future event should be a specific, personal experience that had lasted, or would last, no longer than one day. Participants were instructed to consider as many details of the memory/future event as possible (i.e., what is being done, who they are with, feelings and emotions). Participants were also instructed that a different memory should be used for each cue, although no restrictions on the time frame were made. At the beginning of the task, participants were given instructions with two examples of responses to cue-words for both AMT and EFT-T (See Table S1). The words shown to participants consisted of 12 cues, including six neutral and six negative cues. Due to counterbalancing, participants completed either the AMT1 set paired with EFT2, or the AMT2 set paired with EFT1. Each AMT set contained three neutral and three negative cues, while the corresponding EFT set contained a different set of three neutral and three negative cues.

Participants were presented with three negative cues from either the AMT1 or AMT2 set, depending on the counterbalancing condition, and only responses to these cues were included in the present qualitative analysis; data from the neutral cues and from the EFT tasks were not analysed. The full pool of negative cues consisted of six self- or moral-disgust relevant cue-words (*shame*,* mistrust*,* disloyalty*,* exclusion*,* let down*, and *bullying*) which were evenly distributed across the two AMT sets. Each participant completed one AMT set (AMT 1 or AMT 2) comprising three of these cue-words. For each trial, participants were presented with a cue word displayed on the screen and were given two minutes to generate a written description of a specific event they had personally experienced in the past. Cue words remained visible throughout the response period. The words were chosen that described a violation of one’s personal or socio-moral values or norms and most intensely trigger feelings of disgust [29]. To develop the final cue set, the authors collaborated with a Patient and Public Involvement (PPI) group of native speakers with lived experience of an ED (*n* = 6), and with healthy controls (*n* = 9), to rate a list of negative cue words (betrayal, teasing, mistrust, shunned, exclusion, bullying, mocking, disloyalty, shame, and let down) according to the extent to which each word triggered feelings of disgust towards themselves, others, or the situation, and violated personal, socio-moral values or norms, from 0 (not at all) to 5 (extremely). The PPI group was also asked to generate cues or words relevant to their experience of being disgusted and morally violated. The frequency of ratings was analysed, and six words were selected for inclusion in the task.

### Qualitative analysis

AMs in free-text format (AN = 123 and HC = 60) were condensed and imported into the software program, NVivo version 15, for qualitative data analysis. We used a “conventional qualitative content analysis” approach, which involves inductive coding and is useful when describing a phenomenon about which there is limited existing research [30–32]. In contrast with directed qualitative content analysis that builds on predetermined themes from previous literature, this inductive approach is beneficial as it allows for participants’ accounts to be discussed without the imposition of predetermined ideas. The qualitative analysis was conducted by three coders (SBD, JLK, and FT), all female, psychologists and ED researchers holding PhDs. SBD additionally brings clinical expertise as a standard-level ISST-certified schema therapist, which informed the analytical process. All coders were trained in NVivo, with JLK and FT having prior experience in qualitative research design and analysis. Throughout the analysis, the research team engaged in reflexive discussions, and potentially biases could influence the coding and interpretation of participants’ memories. Building on their combined expertise, the three coders first independently coded the AMs generated in the AN dataset, after which the emerging coding framework was applied to the HC dataset for comparison. The coding frame was then refined to incorporate additional codes that emerged from the HC data. This refinement phase was conducted by two coders, SBD and FT. Subsequently, the two coders revisited the AN dataset to identify any further relevant instances. AMs were segmented into semantic units during coding in NVivo. Consequently, frequencies are reported per memory rather than per semantic unit in the Results section. This approach allows a single memory to be assigned to multiple codes if different segments of that memory correspond to distinct codes, thereby preserving the full narrative and structure of each memory. In the process of developing themes, SBD and FT reviewed each code alongside the corresponding AMs. All codes were visualized using diagrams to facilitate discussion of similarities and connections, and conceptually similar codes were subsequently grouped to form higher-order themes through an iterative process of interpretation and abstraction.

## Results

### Sociodemographic and clinical characteristics

The two groups were comparable in terms of ethnicity, gender, age, and years of education. As expected, however, they differed in self-reported BMI, with the AN group reporting a lower BMI than HCs. Thirteen participants (30%) in the AN group had a BMI ≥ 18.5 kg/m². The proportion of participants with AN currently receiving treatment (e.g., inpatient, outpatient, or private care) was 67.4%. A detailed comparison of socio-demographic and clinical characteristics between groups is presented in Table 1.

**Table 1:** Comparison of socio-demographic and clinical characteristics between anorexia nervosa and healthy controls

	HC (*n* = 20)	AN (*n* = 43)	t or X^2^ (df)	*p* value (d or φ)
Age, years M ± SD	26.60 ± 6.74	28.53 ± 6.97	-1.04 (1, 61)	0.30 (0.28)
Gender, n (%)			2.40 (2)	0.30 (0.19)
Female	17 (85.0%)	40 (93.0%)		
Male	3 (15.0%)	2 (4.7%)		
Non-binary	–	1 (2.3%)		
BMI, kg/m^2^ M ± SD	21.47 ± 2.79	16.98 ± 2.22	8.38 (1, 77)	< 0.001 ** (1.86)
Ethnicity, n (%)			1.02 (2)	0.60 (0.11)
White	17 (85.0%)	40 (93.0%)		
Asian	2 (10%)	2 (4.7%)		
Mixed Race	1 (5.0%)	1 (2.3%)		
Other	–	–		
Years of Education, M ± SD	17.53 ± 2.35	17.51 ± 2.35	0.02 (1, 54)	0.98 (0.01)
Diagnosis Duration (years, M ± SD)	–	10.00 ± 7.75		
Symptom Duration (years, M ± SD)		12.49 ± 8.27		
Comorbidity, n (%)				
Obsessive Compulsive Disorder	–	5 (11.6%)		
Affective Disorder	–	17 (39.5%)		
Anxiety Disorder	–	15 (34.9%)		
Attention Deficit Hyperactivity Disorder	–	1 (2.3%)		
Autism Spectrum Disorder	–	4 (9.3%)		
Current Treatment, n (%)				
Treatment	–	29 (67.4%)		
No Treatment	–	14 (32.6%)		

AN: Anorexia Nervosa; BMI: Body Mass Index; kg/m^2^: kilogram per square metre; HC: Healthy Controls; M: Mean; SD: Standard Deviation; X^2^: Chi-Square; φ: Phi; d: Cohen’s d

*Significant at the *p* < 0.05 threshold

**Significant at the *p* < 0.001 threshold

### Findings of qualitative content analysis

A total of 189 AMs (AN = 129; HC = 60) were included in the qualitative analysis, from which fourteen codes were identified. Of these, as individual memories could encompass multiple semantic units, a subset of AMs (X%) was assigned to more than one code. Two codes, *no recall or experiences* (AN: 5% of AMs; HC: 12% of AMs) and *vicarious experiences* (AN: 4% of AMs; HC: 28% of AMs), were not included in the theme development process. Percentages of memories per code were calculated using the total number of AMs in each group rather than excluding these two codes. Excluding memories would have disproportionately inflated the proportions of AMs for the remaining codes, particularly in the HC group, potentially giving a misleading impression of prevalence. Table 2 presents the frequencies and percentages of participants and memories classified under each code for both groups. These were reported per memory rather than per participant. Participant-level frequencies could obscure the relative prominence of specific codes, as they indicate only whether a code occurred at least once for a given participant, rather than how frequently it appeared across AMs.

Among memories classified under twelve codes, some were observed exclusively in the AN group, including *iatrogenic harm* (3%) and *body mistrust* (2%). Several other codes appeared more often in the AN group, such as *betrayal* (AN: 12%; HC: 2%), *impact of past interpersonal experiences* (AN: 7%; HC: 2%), *moral self-evaluation* (AN: 10%; HC: 5%), *experiences of shame* (AN: 23%; HC: 17%), and *feelings of wrongness or being different* (AN: 5%; HC: 2%). In contrast, *teasing or bullying* (AN = 8%; HC = 12%), *others not meeting expectations* (AN = 15%; HC = 23%), and *ambiguous social exclusion* (AN = 6%; HC = 12%) were more frequently observed in the HC group. Two codes, *interpersonal suspiciousness* (AN = 6%; HC = 7%) and *self-criticism* (AN = 20%; HC = 22%), were observed with similar frequency across both groups.

The above twelve codes were organised into three overarching themes: (1) Relational Wounds – The Role of Others, (2) Relational Vigilance, and (3) Identity Disturbance. Below, each theme and its corresponding codes are outlined, alongside a description of similarities and differences in memory content between groups. Quotations are presented in summarized form rather than verbatim (see ethics declaration).

**Table 2:** Frequencies (percentages) of memories and participants by codes and groups (anorexia nervosa and healthy controls)

Themes	Codes	Description	Frequencies (percentages) of memories		Frequencies (percentages) of participants	
			AN (*n* = 129)	HC (*n* = 60)	AN (*n* = 43)	HC (*n* = 20)
Relational Wounds – The Role of Others	Betrayal	Experiences of betrayal (violation of trust, loyalty, or honesty, or confidentiality, resulting in emotional harm) by close or personal relationship (e.g., friends, family members, romantic partners).	15 (12%)	1 (2%)	13 (30%)	1 (5%)
	Iatrogenic harm	Experiences of harm (emotional, psychological or relational) arises as a result of interactions with healthcare professionals or treatment systems.	4 (3%)	0 (0%)	4 (9%)	0 (0%)
	Teasing or bullying	Experiences where the participant describes being targeted by others through teasing or bullying.	10 (8%)	7 (12%)	9 (21%)	7 (35%)
	Others not meeting expectations	Experiences of daily-life conflicts in which others failed to meet various types of expectations such as broken promises or general disappointments (e.g., not showing up emotionally or physically).	19 (15%)	14 (23%)	18 (42%)	14 (20%)
Relational Vigilance	Ambiguous social exclusion	Experiences where participants feel excluded or left out in social situations, although the intent, reason, or whether the exclusion was deliberate remains uncertain.	8 (6%)	7 (12%)	8 (19%)	7 (35%)
	Interpersonal suspiciousness	Suspicion directed toward close others or strangers, even in the absence of any clear wrongdoing.	8 (6%)	4 (7%)	7 (16%)	4 (20%)
	Impact of past interpersonal experiences	How past negative experiences influence their current behaviours (e.g., avoidance) or attitudes (e.g., mistrust, guardedness).	9 (7%)	1 (2%)	9 (21%)	1 (5%)
Identity Disturbance	Self-criticism	Holding a highly negative attitude toward oneself. This may include feeling inadequacy and being overly critical, an inability to derive satisfaction from one’s behaviour, and chronic concerns about others’ criticism.	26 (20%)	13 (22%)	12 (28%)	12 (60%)
	Moral self-evaluation	Evaluating one’s own actions, intentions, or decisions according to moral, ethical or personal standards, experience moral emotions (e.g., guilt, regret, sense of disloyalty) for perceived wrongdoing or failing to meet moral expectations.	13 (10%)	3 (5%)	12 (28%)	3 (15%)
	Experiences of shame	Feelings of shame occurring in different contexts.	30 (23%)	10 (17%)	25 (58%)	10 (50%)
	Feeling of wrongness or being different	Internal feelings of not belonging, being different/weird/odd, or feeling “wrong” in a particular context or group.	6 (5%)	1 (2%)	5 (12%)	1 (5%)
	Body mistrust	Expressions of mistrust towards the body	2 (2%)	0 (0%)	2 (5%)	0 (0%)
N/A	No recall or experience	No experience of a relevant past event or no recall of any such experience in response to the given cue-words.	6 (5%)	7 (12%)	5 (12%)	5 (25%)
	Vicarious experiences	Events that happened to other people. Participants recalled vicarious memories - experiences involving someone else - rather than first-hand events.	5 (4%)	17 (28%)	5 (12%)	11 (55%)

AN: Anorexia Nervosa; HC: Healthy Controls; N/A: Not Applicable

### Theme 1: relational wounds – the role of others

This theme encompasses memories in which participants reflected on the actions or omissions of significant others, including family, friends, peers, romantic partners, or clinical staff, that contributed to relational injury. These patterns were expressed through four key codes: *betrayal*, *iatrogenic harm*, *teasing or bullying*, and *others not meeting expectations*.

#### Iatrogenic harm

This code emerged exclusively in the AN group. Participants described interactions with clinical staff that, rather than providing support, intensified feelings of being judged, unwanted, or disrespected. One participant described feeling invalidated by a healthcare professional’s comment that framed their distress as a matter of personal choice, which led to increased self-blame and anxiety about future interactions with the staff. Another participant interpreted remarks made within a daycare group stating that certain patients did not belong in the group as personally directed due to their low-weight status, leading to feelings of exclusion. Breaches of trust and confidentiality were also described, such as overhearing staff gossip or a letter from the community mental health team being sent to a participant’s parents’ address instead of their own. Collectively, these experiences demonstrate how the actions or omissions by others involved in a caring capacity during a time of vulnerability (in this case clinical staff or mental health service), may contribute to relational wounds, transforming figures of care into sources of judgment or exclusion.

#### Betrayal

This code appeared solely among AN participants (only one HC memory, reflecting dishonesty, was also coded here). In the AN group, betrayal often involved emotionally significant relationships, particularly romantic partners and family, and was often described as a turning point that altered the course of relationship dynamic or trust. Examples included infidelity, disclosure of confidential information, or being abandoned by significant others during periods of illness or hospitalisation, often resulting in tangible consequences such as separation or estrangement. Some betrayals recalled in the AN group were ED-specific, with participants describing feelings of being betrayed by others in ways directly related to their disorder, for example a parent giving more food than intended, or being abandoned by childhood friends or romantic partner during illness or hospital admissions. Overall, the frequency of such experiences was substantially greater in the AN group, and they predominantly occurred within significant relational contexts rather than more distant or peripheral relationships.

#### Teasing or bullying

This code appeared across both groups and was most commonly situated in school settings. Interestingly, memories in which participants were targeted for physical characteristics, such as weight, or speech, were observed in both groups. Although participants in both groups described these experiences as hurtful, the way these memories were framed appeared to differ. In the HC group, memories more often reflected adaptive reflection or coping, for example recognizing that others’ comments said more about the perpetrator than about themselves, becoming more attentive to early warning signs of potential unfair treatment in others, or feeling more able to assert boundaries in later interactions. These suggest that, while the events were distressing, some individuals in the HC group were able to reappraise them within a broader trajectory of learning or resilience, in contrast to the more enduring sense of injury observed in some accounts from the AN group.

#### Others not meeting expectations

This code was one of the most frequently endorsed codes across both groups. These memories typically involved everyday interpersonal disappointments, such as broken promises, last-minute cancellations, inconsistent behaviour, or failures to provide support during moments of need. Both groups recalled similar types of events; however, in the AN group, memories tended to reflect a prolonged experience of feeling unsupported, misunderstood, or let down by significant others. A few memories specifically described others failing to meet expectations in the context of the ED, such as during hospitalisation or in providing support throughout the illness. In contrast, HC memories tended to reflect greater social insight or perspective-taking; for example, participants recognized that friends might have valid reasons for being unable to attend events, and omissions or disappointments could be openly discussed and resolved, supporting relational stability. By comparison, AN memories more often conveyed persistent feelings of being let down and uncertainty within relationships.

Overall, within the theme of Relational Wounds – The Role of Others, participants with AN recalled relational wounds that were generally more frequent, emotionally intense, and relationally significant, often remaining unresolved, particularly in contexts of betrayal and iatrogenic harm and their EDs. In contrast, controls tended to describe similar past events in more situational or manageable terms, being able to utilise self-protective strategies such as reflection, insight, or empathy to contextualize or resolve experiences of teasing, bullying, or unmet expectations. Across both groups, these memories illustrate the role of interpersonal interactions in shaping trust, a sense of safety, and relational security, highlighting how unresolved relational adversities, especially within close or caregiving relationships, have the potential to leave enduring emotional scars.

### Theme 2: relational vigilance

This theme reflects memories in which participants described heightened alertness, caution, or vigilance toward others in social contexts, linked to prior negative interpersonal experiences. Across memories, participants monitored social cues, questioned others’ intentions, and evaluated the safety or predictability of relationships. These patterns were expressed through three key codes, *ambiguous social exclusion*, *interpersonal suspiciousness*, and *the impact of past interpersonal experiences*.

#### Ambiguous social exclusion

This code highlighted difficulties in interpreting others’ intentions when people were unresponsive, silent, or failed to reply, and such experiences were reported in both AN and HC groups. In the AN group, ambiguous exclusion frequently involved close relationships, such as family or close friends, and often elicited self-doubt or rumination about the reasons for being left out (e.g., questioning whether others did not need or want the participant, or why they had not contacted the participant). This pattern was largely absent in the HC group, whose participants tended to confidently attribute exclusion to group dynamics, or minor misunderstandings, rather than questioning their own relational value.

#### Interpersonal suspiciousness

Building on this heightened sensitivity to social cues, memories in both groups reflected mistrust or caution towards others in various social contexts. Individuals described past events of questioning others’ intentions, feeling uncertain about whether people could be relied upon, doubting the truthfulness or what they were told, being hypervigilant to and monitoring others’ behaviour closely. However, notable differences emerged between the groups. In the AN group, suspiciousness was more pervasive and generalized, extending beyond close relationships to include strangers, professionals, and institutions involved in their care. Some participants reported generalized mistrust toward all professionals who were perceived as vague, while others described difficulty trusting most people or struggling to rely on others to make decisions on their behalf. In contrast, the suspiciousness among controls tended to be more situational and relationship-specific, typically arising in response to discrete events such as a friend cancelling plans, a partner’s unexpected behaviour, or navigating unfamiliar situations alone.

#### Impact of past interpersonal experiences

Memories under this code describes how early relational adversities continued to shape participants’ beliefs about others (e.g., I should never trust anyone; I can’t trust anyone; they will do it again) and the self (e.g., I am in some way not enough of a person) as well as current self-protective attitudes (e.g., guardedness) and behaviours (e.g., eavesdropping conversations for potential deception), often driven by anxiety. These memories typically involved experiences of betrayal, dishonesty, bullying, or broken trust within close relationships (e.g., romantic partners, friends, or family members) and were characterized by their enduring impact on participants’ interpersonal functioning. This code appeared solely among AN participants, although one HC memory was also coded, reflecting a past experience of bullying that the participant described as emotionally painful and continuing to affect.

Overall, within the theme of Relational Vigilance, both groups recalled memories where they experienced some level of relational vigilance; however, among participants with AN, the belief that “others can be unpredictable, unsafe, or disappointing; therefore, I must stay alert” was particularly prominent, extending to longer period of time and multiple relationships or social contexts.

### Theme 3: identity disturbance

This theme reflects autobiographical memories in which participants described a fragmented or unstable sense of self, characterised by difficulties integrating emotional, moral, and bodily dimensions of identity. Across memories, participants grappled with self-evaluative processes that shaped their self-perception, often involving harsh internal judgments, doubts about their own moral integrity, or a felt sense of being fundamentally “wrong,” “different,” or “inadequate.” Together, these patterns were represented across five codes, *self-criticism*,* moral self-evaluation*,* experiences of shame*,* feeling of wrongness or being different*,* and body mistrust*.

#### Self-criticism

All memories under this code reflected the presence of an inner critic, the tone and intensity of which differed between groups. In the AN group, this inner critic was characterised by a markedly shame-laden and self-blaming tone. For example, one participant described this as a way of constantly beating herself up, which prevent her from focusing on her achievements. The criticism tended to target the self at a global level rather than specific actions, with most participants describing themselves as awful, naïve, or stupid. They tended to interpret minor mistakes or interpersonal events as evidence of being fundamentally flawed in a broader sense. The predominant thinking style, characterised by statements such as “I am not good enough,” was reported much more frequently in the AN group than in HCs. This seems to highlight a collapse of the distinction between momentary experiences and core self-definitions, suggesting difficulty maintaining stable boundaries between behaviour and identity. The accounts illustrate how the intensified inner critic contributes to a disrupted, negatively biased, and incoherent self-concept. In contrast, some memories coded under self-criticism in the HC group also reflected self-awareness and adaptive reflection, processes largely absent in the AN group. Even when recalling memories where self-criticism was involved, HCs tended to reappraise these experiences by generating more nuanced and constructive interpretations, thereby rarely implicating their broader self. This included recognizing competing priorities, acknowledging personal strengths, and putting challenges into perspective, suggesting an ability to regulate the inner critic through reframing and, potentially, to maintain a coherent and positive sense of self.

#### Moral self-evaluation

Memories coded under moral self-evaluation also reflected the presence of an inner critic, but one that operates through moral standards, judging the self not for inadequacy, but for perceived violations of integrity, loyalty, or moral duty. A shared feature across both groups was moral self-monitoring, with participants reflecting on whether they had acted rightly or done enough to support others, although the focus and intensity of these evaluations differed markedly between groups. In the AN group, moral self-evaluations were more internalized and closely tied to identity. Participants frequently described concerns about loyalty and disloyalty, both toward significant others and, at times, toward their ED. They often framed everyday situations, (e.g., missing planned activities, deciding whether to visit relatives, discussing family events in therapy, informing flatmates about moving out, reducing or overdoing exercise, lying to a partner, or reacting to a friend’s distress) in moral terms, interpreting them as right or wrong, or as acts of loyalty or disloyalty. These memories reflected an amplified sense of responsibility to act ‘rightly’ (e.g., to be honest, loyal, open, or caring), with participants scrutinising their own behaviours to repair perceived wrongdoings and maintain a sense of moral integrity. Moral self-evaluation contributed to identity disturbance by linking the sense of self to moment-by-moment moral judgments, with individuals ruminating on whether they had done enough for others or not. In contrast, HCs (based on only three memories) tended to describe more situational forms of moral self-evaluation that were not central to their sense of self or moral integrity. Feelings of guilt and senses of responsibility were expressed in a milder tone and did not appear to challenge their overall moral identity. They often focused on everyday lapses in daily routines or social interactions, such as being late, or not helping enough. Importantly, these did not generalise into conclusions about “who I am,” but remained tied to the specific situation, indicating that they reflect moral self-monitoring rather than their identity.

#### Experiences of shame

Memories in the AN group reflected experiences of shame as constant, intense, and enduring. The severity of these experiences was also reflected in responses where shame co-occurred with disgust or self-disgust. Interestingly, one participant described a visceral reaction (gut churn) even seeing the cue word. Furthermore, some individuals reported feeling shame directed at their entire self or at their very existence. In contrast, in the memories of HCs, shame was generally described in milder, more transient terms (e.g., a bit of shame, some shame, slightly shameful). Both groups reported experiences of inadequacy, a core feature of shame; however, in the AN group, these experiences were predominantly tied to ED-specific contexts (e.g., food, eating, exercise, or appearance). Moreover, the disorder itself (including symptoms, illness onset, hospitalization, and recovery processes) appeared to further intensify feelings of shame. In contrast, HCs reported context-specific shame usually tied to social or performance-related situations. These patterns suggest that, in AN, shame functions as a pervasive lens through which both everyday behaviours and broader aspects of self are interpreted. In contrast, among HCs, shame was situational, mainly performance-based, often arising from academic or professional contexts (e.g., sharing exam results with parents, falling behind on the expected progress set by the tutor, or entering the wrong door).

#### Feeling of wrongness or being different

Memories under this code were predominantly reported by individuals with AN, compared with only a single memory from the HC group. Across these memories, participants with AN described their difficulties in interacting with others (mostly friends and sometimes family members, often perceiving themselves as “wrong,” the “odd one out,” or “weird”, which extended beyond momentary or situational experiences. These memories illustrate persistent feelings of social disconnection, loneliness, and a sense of being fundamentally different, and reveal how individuals with AN struggle to understand themselves, maintain a coherent sense of belonging, and navigate their social world with confidence. In contrast, only one memory in the HC group were under this code, for this participant, experiences of feeling different was tied to specific situations or external circumstances, such as behaving differently, without interpreting these differences as reflecting a fundamental flaw in themselves.

#### Body mistrust

This code emerged as the only distinct code under the Identity Disturbance theme, with memories predominantly reflecting ED-specific experiences, such as food restriction and the need for control due to the lack of trust toward the body. This suggests that for individuals with AN, disruptions in the sense of self extend into the embodied domain, linking mistrust of the body to broader struggles with self-coherence.

Overall, within the theme of Identity Disturbance, memories of individuals with AN reflected a lack of coherent identity, characterized by blurred boundaries between behaviour and the core self. This often led to persistent confusion and self-doubt, a pervasive sense of being not good enough or defective, and pervasive and all-encompassing experiences of shame. In contrast, HCs generally experienced situational, flexible, and adaptive self-evaluations that supported a coherent and stable sense of self, even when they reported shared experiences such as self-criticism, moral self-monitoring, or shame.

## Discussion

The present study employed inductive content analysis to examine similar and/or divergent patterns in AMs (total *n* = 189; AN *n* = 129; HC *n* = 60) among individuals with AN compared to HCs. Ten codes contributed to the development of three overarching themes: (1) relational wounds – the role of others, (2) relational vigilance, and (3) identity disturbance. Of the codes, two, *iatrogenic harm* and *body mistrust*, were observed exclusively in the AN group. Beyond these exclusive codes, five codes, *betrayal*, *impact of interpersonal experiences*, *moral self-evaluation*, *experiences of shame*, and *feelings of wrongness or being different*, were more frequently represented in the AN group. In contrast, three codes, *teasing or bullying*, *others not meeting expectations*, and *ambiguous social exclusion*, were more frequently observed in the HC group. Finally, two codes, *interpersonal suspiciousness* and *self-criticism*, appeared comparably across both groups. Overall, individuals with AN exhibited more intense, enduring, and unresolved relational and self-related difficulties whereas HCs described similar experiences in a more situational, flexible, and adaptive manner. It is important to note that experiences within the AN group were heterogeneous, with no single code endorsed by all participants (see Table 2); accordingly, the memory content presented under each of the three overarching themes represents dominant patterns rather than universal experiences.

The theme “Relational Wounds – The Role of Others” encompassed memories in which significant others acted as sources of harm or neglect. Experiences of betrayal and iatrogenic harm were either unique to or more frequently reported by individuals with AN, often arising from family members or interactions with clinicians or the broader healthcare system. For people with AN, who may experience significant social isolation, this is ever more pertinent given that these individuals may comprise their entire social network [33–35]. These relational injuries were also closely tied to ED contexts in the AN group, including unmet expectations during daily routines or treatment-related experiences. Notably, experiences of betrayal were largely absent among HCs, who instead described other adversities, such as teasing, bullying, or minor disappointments, in more situational and manageable terms, often employing reflection, insight, or empathy to contextualize or resolve these experiences. These findings support evidence that individuals with AN may show increased sensitivity to social rejection [19], difficulties with emotion regulation [36] and with perspective-taking [37], potentially arising from early emotional neglect or trauma, which can compromise socio-emotional functioning—consistent with recent research [38]. Importantly, these findings extend prior research on adverse childhood experiences (ACEs) [39] by highlighting the salience of both childhood and adulthood adversities in people with AN.

Relatedly, AN participants recalled memories characterized by heightened alertness and careful monitoring in social interactions, particularly with others perceived as unpredictable or unreliable, a pattern captured under the theme “Relational Vigilance.” Between-group differences were most pronounced in the code *impact of interpersonal experiences*, with individuals with AN exhibiting greater learned guardedness and persistent mistrust across multiple relationships and social contexts. From the attachment theory perspective, both themes may reflect attachment insecurities (of both anxious or avoidant varieties) that are commonly observed in AN [40, 41], developing or strengthening the belief that others are unpredictable or unsafe. Social learning processes [42] may further reinforce these core schemes or beliefs, whereby repeated exposure to attachment wounds leads to threat expectancy and/or monitoring especially in ambiguous situations. Although vigilance can serve as an adaptive function in predicting or preventing harm in AN; it may become generalized and chronic, potentially intensifying fear of trusting others and hindering the development or maintenance of close relationships, thereby evolving into a maladaptive pattern that may aggravate AN psychopathology via isolation. Findings from a recent systematic review and meta-analysis of 47 studies [19] showed that patients with AN exhibit a cognitive profile that could lead to a tendency to expect rejection, readily perceive rejection and react with more intense negative affect to rejection-based cues. Notably, authors explicitly highlighted an absence of studies investigating episodic memories of prior social rejection experiences. The pattern of vigilance observed across AMs in our study may reflect heightened social rejection sensitivity operating at the level of episodic memory content. Also, our prior study showed that AMs elicited by self- or moral disgust relevant cues were recalled more vividly in individuals with EDs who reported greater prior victimization; this pattern was not observed in the HC group [13]. Vivid recall may increase the perceived relevance of past interpersonal experiences for interpreting current relationships, potentially through heightened vigilance to social threat, reflecting a maladaptive pattern of schema-driven information processing [43].

Importantly, AM content did not only reflect relational wounds or relational vigilance but also revealed differences in identity-related experiences, characterised by persistent self-doubt, lack of trust towards body, feelings of defectiveness, and chronic shame in the AN group. The theme “Identity Disturbance” can be understood in light of schema-mode models [44], in which a harsh and punitive inner critic plays a central role in shaping self-concept. This was reflected in two codes, *self-criticism* and *moral self-evaluation*. Interestingly, moral self-evaluation appeared more frequently in the AN group, suggesting that the inner critic operates not only through global negative self-judgment but also through rigid moral standards (e.g., being honest, loyal, open, or caring) and heightened sensitivity to perceived failures of loyalty, responsibility, or moral correctness. Such moralised forms of self-scrutiny align with prior research indicating that individuals with AN often anchor their sense of worth in being “good,” “loyal,” or otherwise morally adequate such that even minor interpersonal or behavioural lapses can be experienced as profound threats to the self [45]. Thus, individuals often reported seeing themselves as fundamentally “wrong” as exemplified in the code *feelings of wrongness or being different.* In contrast, the few memories of moral self-evaluation observed in HCs were more situational and were not central to their sense of self or moral integrity.

Although the overall frequency of memories coded as *self-criticism* was comparable between-groups, shame-driven forms of criticism were far more prominent in the AN group. Moreover, *experiences of shame* in the AN group were not transient or context-bound, as they tended to be for HCs, but rather chronic and pervasive. While shame was often expressed within ED-specific contexts, experiences associated with having an ED appeared to function as an additional and salient source of shame. This shame reflected experiences of stigma and self-stigma, as well as the experiences of deindividuation and loss of personal agency that often comes with experiencing AN. ED symptoms or treatment-related experiences were described as intensifying shame, and in some cases, participants described experiences of disgust or self-disgust, consistent with the findings of Bektas et al. [15]. Alongside the code of *experiences of shame*, the code of *feelings of wrongness or being different* also emerged, and together, these codes closely mirror the defectiveness/shame schema, which has been identified as a disorder-specific schema in individuals with AN in previous research [46–49]. Consistent with this early maladaptive schema, individuals tend to view themselves as fundamentally flawed, unlovable, disgusting, or inherently inadequate. Prior qualitative research has suggested that AN can become closely intertwined with an individual’s identity and sense of self, with weight loss serving to produce a sense of achievement and manage underlying anxieties [50]. Extending this literature, our findings indicate that individuals with AN also experience pervasive shame and feelings of defectiveness that are frequently activated in daily life. When combined with limited adaptive emotion regulation strategies, these identity-related vulnerabilities may contribute to the maintenance of AN psychopathology, as also suggested in a narrative review by Croce et al. [51]. In this sense, ED behaviours, such as restrictive eating, may function as maladaptive coping mechanisms that temporarily increase a sense of self-efficacy, yet over time reinforce the underlying shame and feelings of defectiveness. In contrast, memories from HCs suggest that, even when experiencing shame or self-criticism, they are more able to externalize related events, preventing them from defining their sense of self or identity.

The sociodemographic and clinical characteristics of the AN sample are relevant for the interpretation of the three themes. For example, treatment status may be particularly relevant to the prominence of iatrogenic harm-related memories: all individuals coded in this category reported being in active treatment. They may therefore be more likely to have had recent treatment-related relational experiences, which could result in a greater proximity and salience of these experiences at the time of memory retrieval. Illness duration may also shape the content of memories captured under the themes of relational vigilance and identity disturbance. With a mean illness duration of 10 years (SD = 7.75), the present sample reflects a longstanding, though variable, course of AN; it may be the case that the longer the illness persists, the more opportunities there are for relational adversities to accumulate and for maladaptive core beliefs about the self and others to consolidate [44], potentially intensifying the chronic and pervasive quality of vigilance and identity-related difficulties observed in the AN group. Furthermore, BMI may be closely relevant to the codes of body mistrust and experiences of shame: lower BMI has been associated with poorer interoceptive awareness [52], which may deepen the disconnection from, and distrust of bodily experience captured under the body mistrust code, while also amplifying shame and, in more severe cases, self-disgust. Nevertheless, these interpretations should be considered tentative, as the present study was not designed to directly examine the influence of these variables on memory content. Future research is needed to empirically test these hypotheses.

### Strengths and limitations of the present study

A key strength of the present study is its use of a qualitative approach to compare AMs between individuals with AN and HCs; to our knowledge, no prior research has explored the phenomenology of AN through the analysis of AM content. There are, however, several limitations to this study. First, there were ethnic/racial differences between the groups, as the HC group was more diverse due to recruitment from the KCL community, which predominantly included students living in the UK for postgraduate education. Since it was not possible to recruit participants from diverse backgrounds in the ED group, we had to exclude sixteen HC participants to match the groups in terms of ethnicity. While this improved comparability, it reduced the relevant content and may have affected the representativeness of the data. Second, participants generated AMs in response to given cue words within the two-minute AMT task, which inherently limited the richness and contextualisation of the data. Unlike in-depth semi-structured interviews, this method did not allow for clarification or elaboration of ambiguous responses, and the brevity of the narratives increases the risk that individual memories could be interpreted out of context. Third, two codes, *no recall or experience* and *vicarious experiences*, emerged but were not included in theme development. As a result, there was naturally less data available from the HC group for qualitative comparison, since a substantial proportion of their memories (28%) were coded as *vicarious experiences*, compared with only 4% in the AN group. Future research could investigate the functional role of vicarious memories, particularly their potential contributions to empathy, perspective-taking, or prosocial behaviour [53], as well as examine whether the lower prevalence of such memories in individuals with AN might re-emerge, and, if so, what factors might explain such patterns. Fourth, although screening calls were conducted by a qualified psychologist, these calls did not constitute a formal structured diagnostic interview, and reliance on participant-reported diagnosis alongside the EDDS as a self-reported screening measure represents another limitation of the current study. Fifth, although participants were provided with instructions to complete the study in a quiet and undisturbed environment, they were not asked to confirm the location in which they completed the tasks. As such, it is not possible to verify whether all participants adhered to these guidelines, which may have introduced variability in the testing environment across participants. Future research could incorporate quality control questions to assess whether participants completed the study under the instructed conditions and remained attentive throughout the tasks. This would help improve data reliability and reduce potential variability arising from uncontrolled testing environments.

### Clinical implications

The current study has several key implications. First, the emergence of the *iatrogenic harm* code aligns with the previous literature documenting unintentional physical, mental or emotional illness or injury acquired by experienced by patients with AN within medical care, both inpatient and outpatient [54–57]. This is a highly critical and urgent issue necessitating systemic change. By implementing trauma-informed approaches in ED services that prioritize safety, trust, collaboration, and non-judgmental communication, the care environment can become safer for patients, enabling them to engage more effectively in treatment and, importantly, reducing the risk of exacerbating their symptoms. These findings might also indicate an increased sensitivity compared to non-clinical sample. Therefore, fostering emotional resilience might benefit people with AN clinically. Additionally, clinicians may be some of the only people in regular contact with patients with AN, because people with AN often experience significant social isolation [34]. This may be a challenging aspect of treatment, particularly given the high levels of social anxiety reported in patients [58], which may limit engagement in broader social relationships. Therefore, interventions that address loneliness and actively promote safe and supportive social connections may represent important components of treatment.

Greater attention to AMs may help to facilitate a coherent sense of self or reintegration of self in people with AN, for instance, through treatment approaches such as Eye Movement Desensitization and Reprocessing (EMDR) or Imagery Rescripting (ImRs) [59]. Both approaches aim to alter maladaptive information (including thoughts, sensations, and emotions) linked to distressing or traumatic experiences. EMDR employs eye movements during the recall of aversive or traumatic memories, which in turn reduces the vividness and emotional intensity of the memory [59]. On the other hand, ImRs is aimed to alter the meaning and emotional response of the memory by encouraging patients to reappraise the meaning of memories through adopting new (more positive or realistic), perspectives when encountering aversive events [60, 61]. ImRs is one key cognitive-experiential technique extensively used in Schema Therapy [43, 62], focusing on addressing the child’s unmet needs and modifying the dysfunctional meanings associated with the memory. Recently, ImRs has begun to be applied to individuals with EDs or disordered eating. Although studies by Zhou et al. [63, 64] provide preliminary support for its effectiveness in people with EDs, findings within clinical samples remain inconsistent and reductions in psychopathology are small-sized, highlighting the need for further research, particularly in people with AN, to establish the efficacy of ImRs in this population. Together, these findings highlight the need to enhance treatment approaches that more directly address AMs. Another potential adjunct is diary writing, particularly during recovery, which could help individuals with AN record, reflect on, and process emotionally salient AMs in collaboration with the therapist. This approach has been discussed by Alexander [65] in terms of its potential benefits and pitfalls.

### Conclusion

This study utilised content analysis to explore the content of self- or moral-disgust-cued autobiographical memories, identifying themes relating to interpersonal and self-directed mistrust, as well as a fragmented identity, and defectiveness shame in people with AN. Autobiographical memory enquiry may constitute a useful tool to facilitate additional insights into the origins of the individual’s problems (i.e., bolster psychological formulation) and their effects on the current presenting problem, as well as the individual’s view of themselves, the world and interpersonal interactions; yet the content and clinical relevance of such memories are likely to vary meaningfully across individuals. Future studies with larger samples should examine how sociodemographic and clinical variables such as age, treatment status, and illness duration and severity shape autobiographical memory content in AN, findings that could in turn inform how memory-based interventions are tailored to individual clinical profiles.

## Electronic Supplementary Material

Below is the link to the electronic supplementary material.

Supplementary Material 1.

## Data Availability

The original dataset, which includes verbatim participant quotations, cannot be publicly shared due to the lack of explicit participant consent for data sharing in the present study.
