# Supplementary material for: Interpersonal wounds and identity among people with anorexia nervosa: a qualitative content analysis of autobiographical memories elicited by disgust-related cues
Source: J Eat Disord. 2026 Jun 8;14:128. doi: 10.1186/s40337-026-01676-w (PMC13248323; doi:10.1186/s40337-026-01676-w)
Supplement: Supplementary file 1 — Supplementary Material 1. [file 40337_2026_1676_MOESM1_ESM.docx]

**Supplementary Information**

**Additional file 1.**

**Table S1.** Autobiographical memory test instructions

| **Autobiographical Memory Task** |
| --- |
| You will now be presented with a series of 6 words, one at a time. Your task will be to describe a specific event that happened to you in the past (from a week ago or longer). The event does not have to be directly related to this word but may be just inspired by it. Here are some instructions for what type of past event to describe:   - It should be something you were personally involved in - It should be something that occurred in a particular place and within the space of a day at most. - Please try and state as many details about this past event as you can (e.g. where it happened, who was there, what happened, how you felt, what you thought etc.). - Please describe a different event for each word.   You will be given 2 minutes to respond. You will then be asked to rate this memory on a series of scales. Please click continue for some examples of responses to this task |
| If the word was “UPSET”, then an example of a response would be:  A few weeks ago, my partner and I had a big fight. We were at home, and we had a disagreement about the housework. I felt very stressed and sad and went for a walk to take my mind off it. I remember thinking that the relationship was not great at that time.  If the word was “HOUSE”, then an example of a response would be:  Last week I went to view a house, as I am thinking about moving. It was in the perfect location for work and looked really nice inside, but it is slightly out of my budget. I felt apprehensive about making any decisions on whether to put an offer in and thought I should wait and see what else comes up on the market. |
| Please press the button below to continue to the task. You will have 2 minutes per word to write your response. Remember to try and state as many details about this past event as you can (e.g. where it happened, who was there, what happened, how you felt, what you thought etc.). Please describe a different event for each word. |
